# An Anilinoquinazoline Derivative Inhibits Tumor Growth through Interaction with hCAP-G2, a Subunit of Condensin II

**DOI:** 10.1371/journal.pone.0044889

**Published:** 2012-09-13

**Authors:** Hirokazu Shiheido, Yuhei Naito, Hironobu Kimura, Hiroaki Genma, Hideaki Takashima, Mayuko Tokunaga, Takao Ono, Tatsuya Hirano, Wenlin Du, Taketo Yamada, Nobuhide Doi, Shiro Iijima, Yutaka Hattori, Hiroshi Yanagawa

**Affiliations:** 1 Department of Biosciences and Informatics, Keio University, Kohoku-ku, Yokohama, Japan; 2 Clinical Physiology and Therapeutics, Faculty of Pharmacy, Keio University, Minato-ku, Tokyo, Japan; 3 Chromosome Dynamics Laboratory, RIKEN Advanced Science Institute, Wako, Saitama, Japan; 4 Department of Pathology, School of Medicine, Keio University, Shinjuku-ku, Tokyo, Japan; Biological Research Centre of the Hungarian Academy of Sciences, Hungary

## Abstract

We screened 46 novel anilinoquinazoline derivatives for activity to inhibit proliferation of a panel of human cancer cell lines. Among them, Q15 showed potent *in vitro* growth-inhibitory activity towards cancer cell lines derived from colorectal cancer, lung cancer and multiple myeloma. It also showed antitumor activity towards multiple myeloma KMS34 tumor xenografts in lcr/scid mice *in vivo*. Unlike the known anilinoquinazoline derivative gefitinib, Q15 did not inhibit cytokine-mediated intracellular tyrosine phosphorylation. Using our mRNA display technology, we identified hCAP-G2, a subunit of condensin II complex, which is regarded as a key player in mitotic chromosome condensation, as a Q15 binding partner. Immunofluorescence study indicated that Q15 compromises normal segregation of chromosomes, and therefore might induce apoptosis. Thus, our results indicate that hCAP-G2 is a novel therapeutic target for development of drugs active against currently intractable neoplasms.

## Introduction

Although advances in treatment, such as combination chemotherapy and chemoradiation, have slightly improved the outcome of tumor therapy over the last several decades [1], tumors are the leading cause of death in economically developed countries and the second leading cause of death in developing countries [2]. Colorectal tumors, lung tumors, and multiple myeloma (a hematopoietic tumor) are particularly intractable. Therefore, novel drugs having potent activity are required to treat such tumors, and in order to develop them, it is important to identify novel molecular targets related to the pathogenesis of these intractable tumors.

**Table 1:** IC_50_ values (µM) of anilinoquinazoline derivatives for inhibiting proliferation of different human tumor cell lines.

Cell lines	Compounds		
	Q15	Q16	Q17
KMS11	5.1	13.5	10.1
KMS27	14.5	19.1	20.9
KMS34	1.1	5.8	6.1
KMM1	4.7	22.1	20.3
RPMI8662	2.3	22.4	6.1
SW480	2.0	6.4	17.4
HeLa	3.3	7.3	13.5

Anilinoquinazoline derivatives such as gefitinib and erlotinib, selective tyrosine kinase inhibitors, have been reported to be effective against recurrent non-small-cell lung tumor [3], [4]. Here, we screened 46 anilinoquinazoline derivatives, which are structurally similar to gefitinib or erlotinib, for growth-inhibitory activity towards a panel of intractable tumor cell lines. Among these compounds, we identified Q15 as a potent proliferation inhibitor and apoptosis inducer of the colon tumor, lung tumor and multiple myeloma cell lines examined. We further confirmed that Q15 showed higher antitumor activity than gefitinib towards multiple myeloma KMS34 tumor xenografts in lcr/scid mice *in vivo*. Surprisingly, however, Q15 did not inhibit intracellular signaling or the phosphorylation status of ERK1/2, indicating that the mechanism of its antitumor effect is different from that of gefitinib. Therefore, we next focused on the possible mechanism of action of Q15.

Previously we have developed an mRNA display system named *in vitro* virus (IVV) [5]–[7], in which an *in vitro*-translated full-length protein (phenotype) is covalently attached to its encoding mRNA (genotype) through puromycin [8]. Here, we employed this mRNA display method to search for the target of Q15 for induction of apoptosis, and identified hCAP-G2, which is a subunit of condensin II [9], [10], as a Q15 binding partner. We further confirmed that Q15 binds to the condensin II complex through direct interaction with hCAP-G2, and therefore may affect chromosomal segregation in mitosis, resulting in abnormal cell division and subsequent apoptosis. These results indicate that Q15 is a promising candidate drug for treatment of high-risk multiple myeloma. Further, hCAP-G2 appears to be a novel therapeutic target for development of drugs active against currently intractable neoplasms.

**Figure 1:**
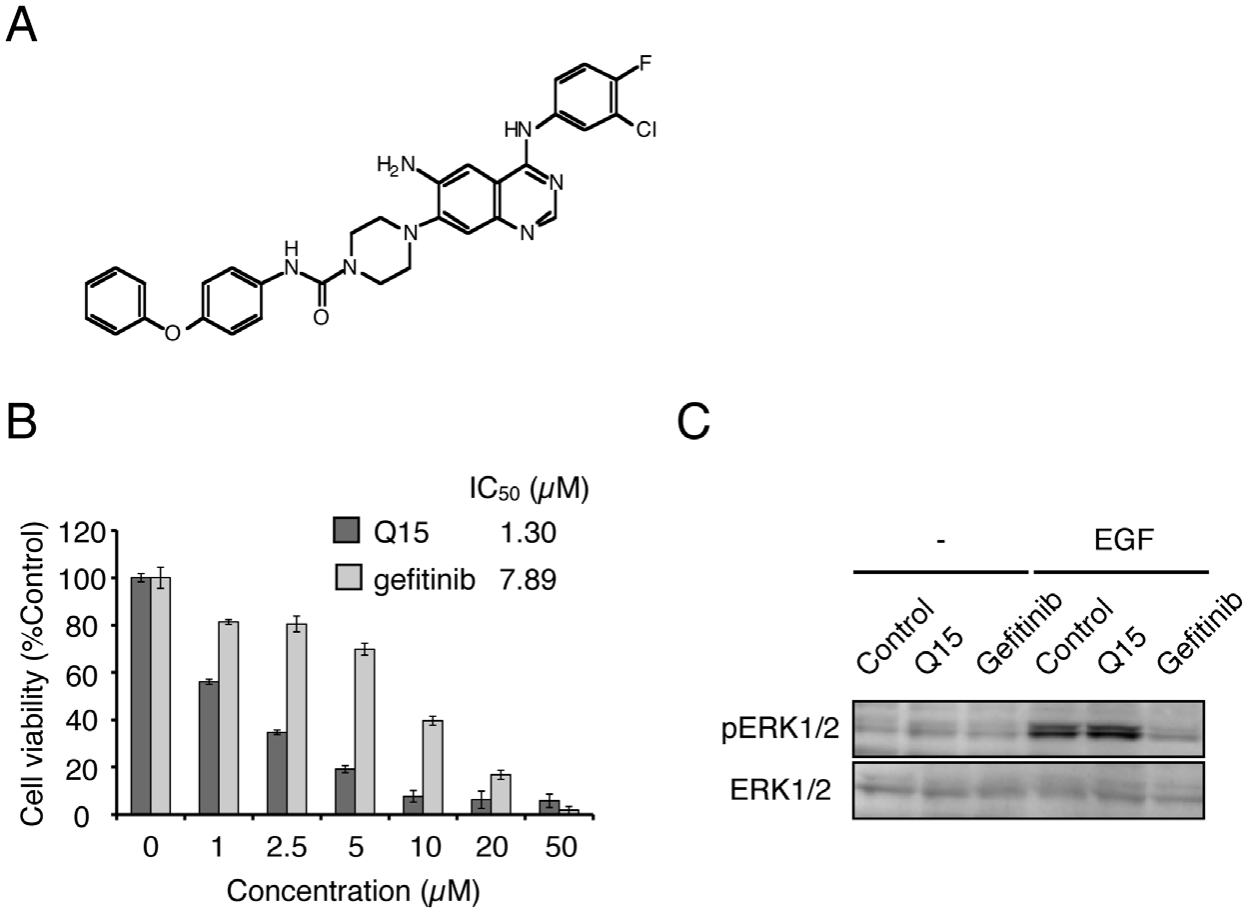
Q15 inhibits proliferation of tumor cells through a different mechanism from that of gefitinib. (A) Chemical structure of Q15. (B) KMS34 cells were incubated with 0–50 µM Q15 or gefitnib for 72 h. Then, cell viability was determined by means of MTS assay. (C) A549 cells were incubated with DMSO or 5 µM Q15 for 24 h in the absence or presence of 50 ng/mL EGF for 48 h. Whole cell lysates were analyzed by Western blotting with anti-pERK1/2 or anti-ERK1/2 antibody.

## Results

### Identification of a Potent Proliferation Inhibitor of Several Multiple Myeloma Cell Lines from a Library of Anilinoquinazoline Derivatives

We screened a compound library consisting of 46 anilinoquinazoline derivatives for activity to inhibit proliferation of various sorts of intractable tumor cell lines. Five multiple myeloma cell lines (KMS11, KMS27, KMS34, KMM1 and RPMI8662), SW480 cells and HeLa were treated with 0.5–50 µM of each compound for 72 h and their viability was examined by means of MTS assay (Table 1). Q15 (Fig. 1A) showed the highest cytotoxicity among the compounds examined towards the cell lines tested here. Next, we compared Q15 with gefitinib in a KMS34 cell proliferation assay (Fig. 1B). The results indicated that Q15 is 6 times more potent than gefitinib.

In order to see whether the mechanism of action of Q15 differs from that of gefitinib, we examined inhibition of phosphorylation of ERK1/2, which is a key growth transducer of tumor cells, by Q15 and gefitinib. A549 cells were treated with 5 µM Q15 or gefitinib for 24 h in the absence or presence of EGF, then subjected to immunoblot analysis. Gefitinib inhibited the phosphorylation of ERK1/2, but Q15 did not (Fig. 1C). Furthermore, Q15 did not inhibit the phosphorylation stimulated by FGF2, HGF, VEGF or IL-6 (data not shown). Thus, these results indicate that the mechanism of action of Q15 is different from that of gefitinib, despite the structural similarity between the two compounds.

**Table 2:** IC_50_ values (µM) of Q15 for inhibiting proliferation of different human tumor cell lines.

Cell Lines	IC_50_ (µM)	Cell Lines	IC_50_ (µM)
Breast cancer		Lung cancer	
HBC−4	2.2	NCI-H23	4.4
BSY-1	3.0	NCI-H226	3.1
HBC-5	3.1	NCI-H522	0.4
MCF-7	3.4	NCI-H460	1.7
MDA-MB-23	1.6	A549	1.3
Brain cancer		DMS273	1.3
U251	1.8	DMS114	1.7
SF-295	1.3	Kidney cancer	
SF-539	2.3	RXF-631L	1.8
SNB-75	1.6	Gastric cancer	
SNB-78	3.1	St-4	1.7
Colon cancer		MKN1	3.1
HCC2998	2.2	MKN7	1.6
HT-29	0.6	MKN28	2.7
HCT-15	1.7	MKN45	1.8
HCT-116	2.4	MKN74	1.4
Ovary cancer		Melanoma	
OVCAR-4	2.3	LOX-IMVI	0.4
SK-OV-3	2.9		

We next tested the antitumor activity of Q15 against several human cancer cell lines, including breast, brain, colon, lung, ovary, kidney, gastric cancer and melanoma (Table 2). Q15 inhibited the growth of all the tumor cell lines examined, suggesting that it may have activity against a wide range of human cancers.

### Q15 Inhibits Cell Proliferation of Multiple Myeloma and Induces Apoptosis Both *in vitro* and *in vivo*

**Figure 2:**
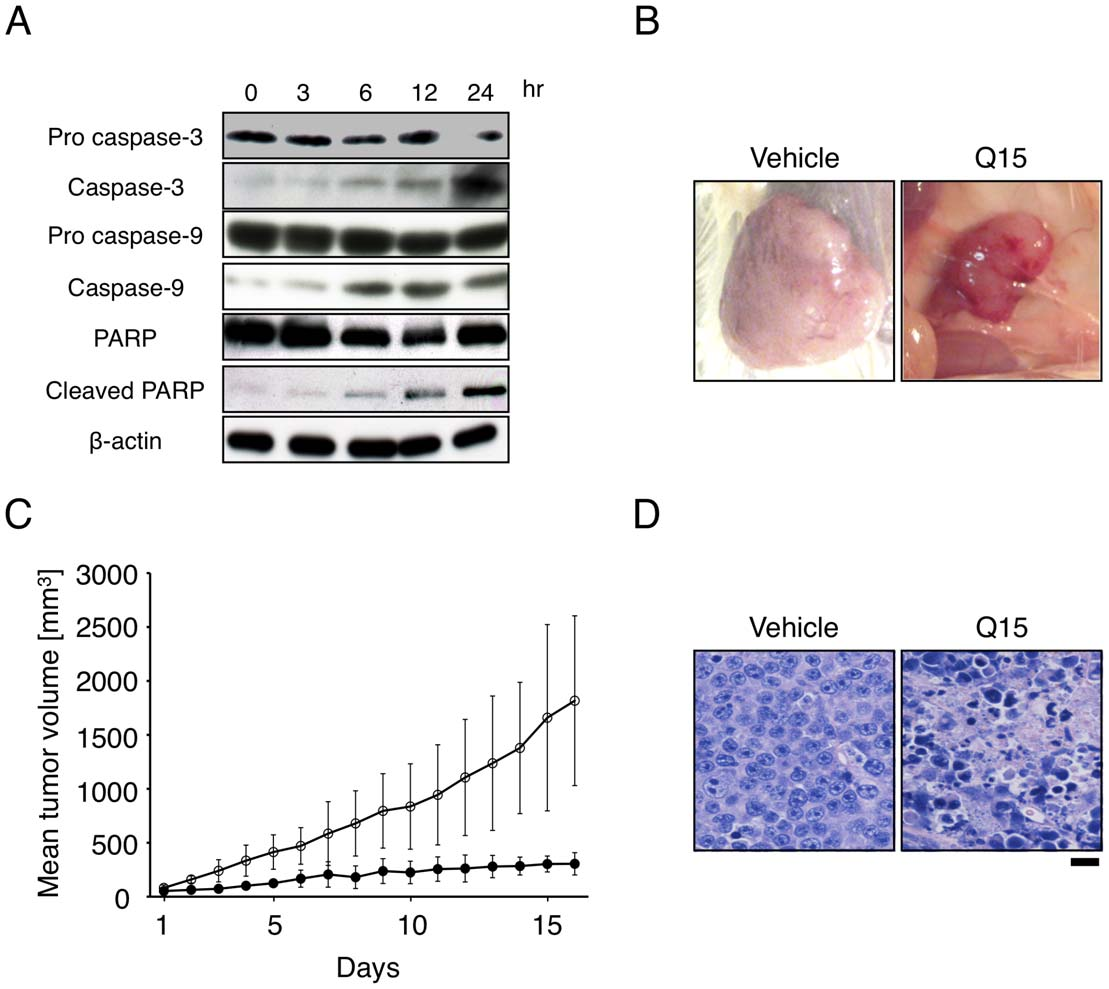
Q15 suppresses tumor growth and induces apoptosis of multiple myeloma cells *in vitro* and *in vivo*. (A) KMS34 cells were incubated with 20 µM Q15 for 0–24 h. The whole cell lysates were analyzed by immunoblot with anti-caspase-3, caspase-9, PARP or β-actin antibody, respectively. (B) KMS34 cells (3×10^7^ cells) were inoculated subcutaneously into lcr/scid mice. Then, 20 mg/kg Q15 was injected intraperitoneally twice every 3 days. Representative tumor from each group. (C) The width and length of the plasmacytoma were measured and tumor volume was calculated. (D) Sections were stained with hematoxylin and eosin.

We next examined the ability of Q15 to induce apoptosis of tumor cells. KMS34 cells were treated with 20 µM Q15 for 0–24 h and then immunoblot analysis of the whole cell lysates was performed. Activation of caspase-3 and 9, leading to cleavage of PARP, was detected (Fig. 2A). These results suggest that Q15 induces caspase-dependent apoptosis of tumor cells.

To examine whether Q15 exhibits antitumor activity *in vivo*, we performed *in vivo* tumor proliferation assay. KMS34 tumor xenografts were treated with intraperitoneal injection of 20 mg/kg of Q15 twice, with a three-day interval, and then the time-course of tumor volume was followed for 16 days (Fig. 2B and C). On the 16th day, Q15 achieved statistically significant inhibition of tumor growth (P<0.02). No significant change of body weight was observed in Q15-treated mice, suggesting that systemic toxicity of Q15 is likely low. In order to examine whether Q15 induces apoptosis of tumor cells *in vivo*, we also performed histopathological examination of tumor tissue. In Q15-treated mice, a number of tumor cells exhibited aggregation of chromatin, as compared with the control (Fig. 2D). This result indicates that Q15 induces apoptosis of tumor cells *in vivo*, as well as *in vitro*.

**Figure 3:**
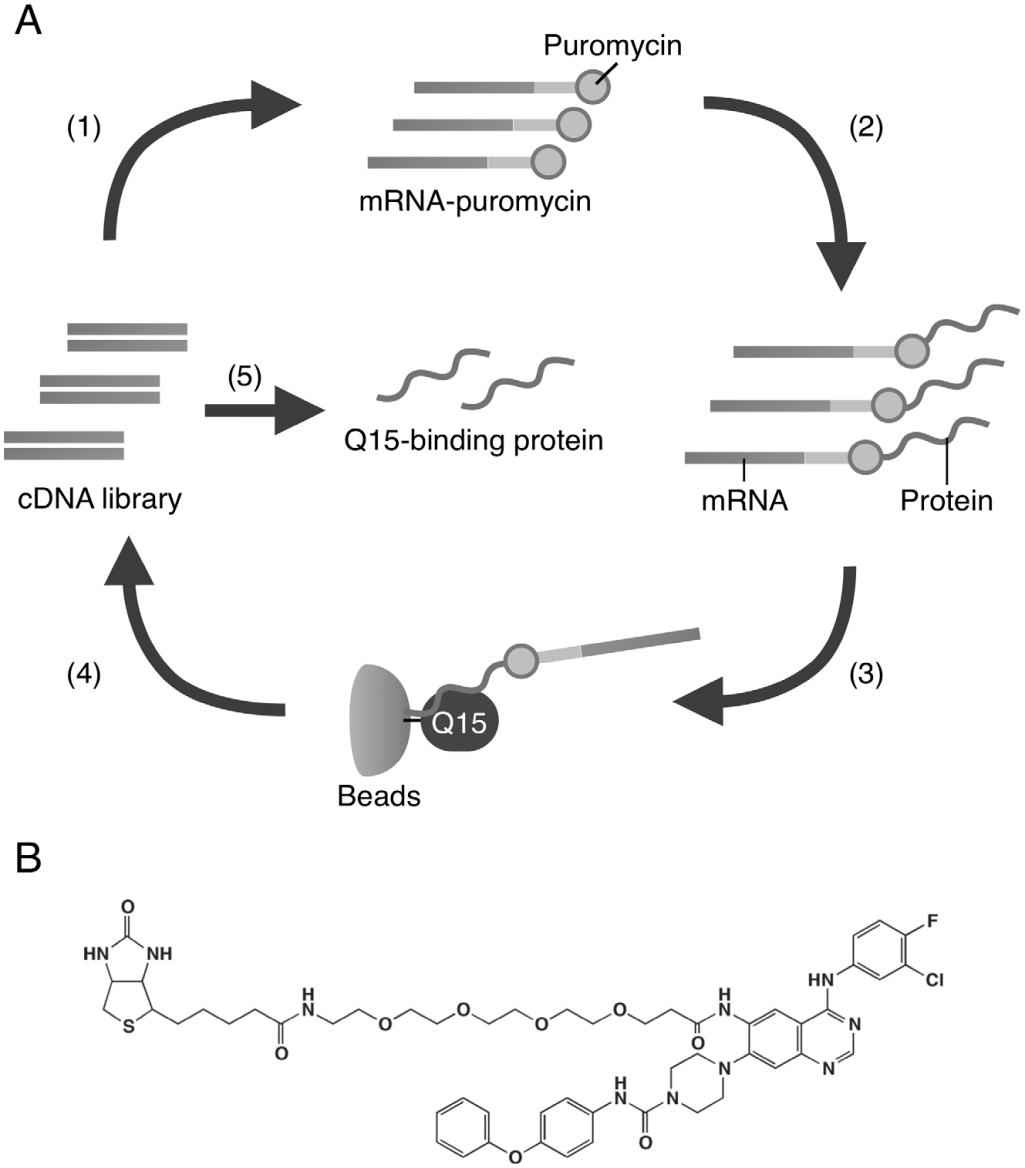
Schematic representation of in vitro selection of Q15-binding protein by mRNA display. (A) A cDNA library derived from SW480 cells was transcribed, ligated with PEG-Puro spacer (1) and translated in vitro (2) to form a protein-mRNA conjugates library. The library was incubated with biotinylated Q15-immobilized beads (3) and unbound molecules were washed away. The bound molecules were eluted and their mRNA portion was amplified by RT-PCR (4). The resulted DNA was used for the next round of selection and analyzed by cloning and sequencing (5). (B) The chemical structure of biotinylated Q15.

**Figure 4:**
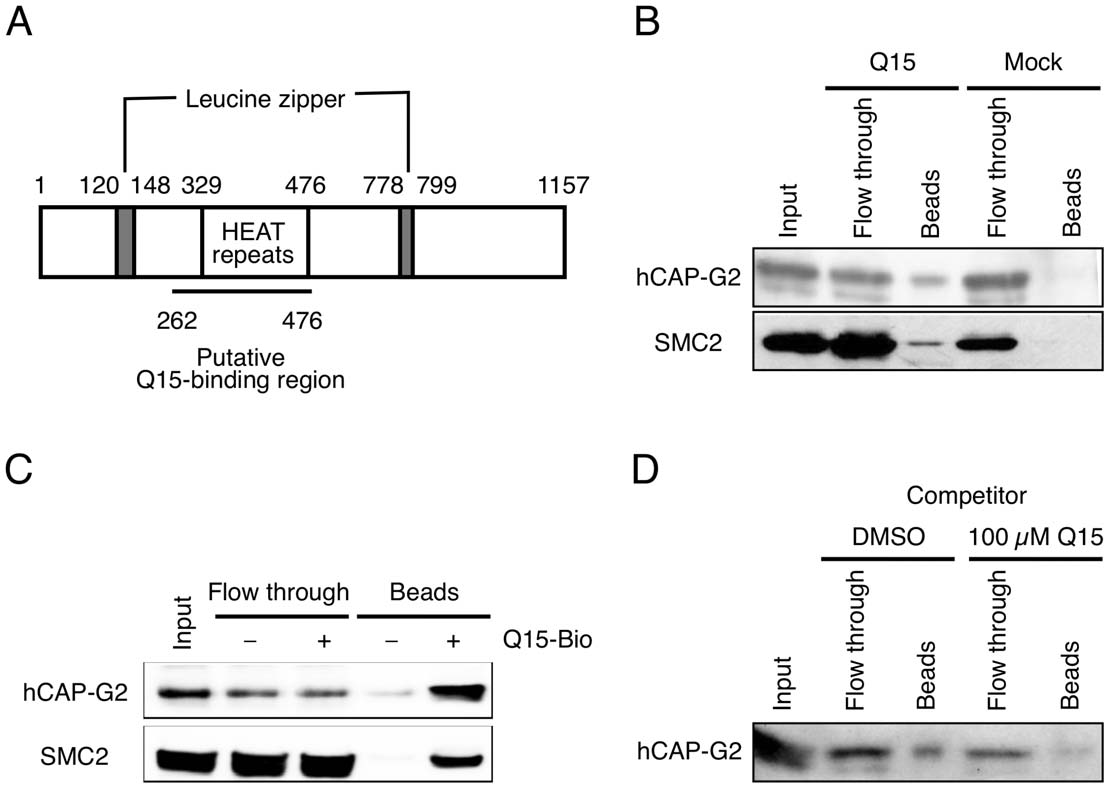
hCAP-G2 protein interacts directly with Q15. (A) Domain structure of hCAP-G2. The region (262–476) selected by mRNA display is underlined. (B and C) Lysates from KMS34 (B) and SW480 (C) cells were incubated with Q15-immobilized beads for 2 h. Each fraction was subjected to 10% SDS-PAGE. Protein bands were detected by immunoblotting with antibodies against hCAP-G2 and SMC2. (D) Cell lysates from KMS34 cells were incubated with Q15-immobilized beads for 2 h in the absence or presence of 100 µM free Q15. Each fraction was subjected to 10% SDS-PAGE and protein bands were detected by immunoblotting with antibodies against hCAP-G2.

### Identification of Q15-binding Protein using mRNA Display

To elucidate the mechanism through which Q15 inhibits proliferation of tumor cells, we set out to identify Q15-binding proteins by means of mRNA display [5]–[7], as illustrated in Fig. 3A. We prepared a cDNA library derived from total RNA of human colon carcinoma SW480 cells, because, like other tumor cells, SW480 cells were sensitive to Q15. Proteins that bind to Q15-Bio (Fig. 3B) immobilized on beads were selected using mRNA display. From the library obtained after 5 rounds of selection, we analyzed the DNA sequences of 100 clones. Among them, we obtained six clones of a fragment of the Luzp5/NCAPG2 gene encoding hCAP-G2_262–476_ containing the HEAT (Huntingtin, elongation factor 3, a subunit of protein phosphatase 2A, TOR lipid kinase) repeat domain (Fig. 4A). Although three other clones were obtained redundantly, they were confirmed to be false-positive clones by means of binding assay (data not shown). hCAP-G2 is a subunit of condensin II complex, which is regarded as a key player in mitotic chromosome condensation [9], [10], [11].

**Figure 5:**
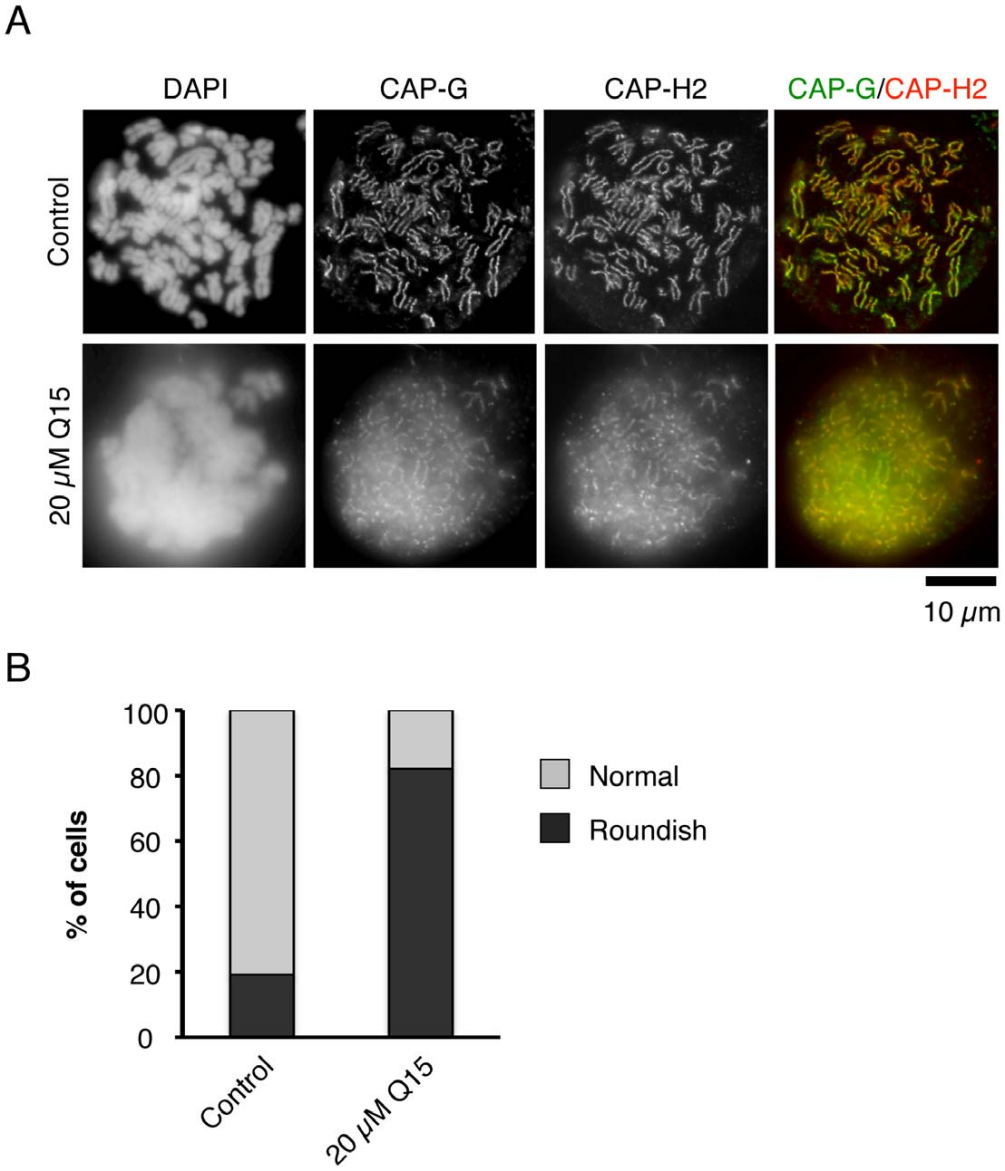
Q15 induces structural aberration of chromosomes in mitosis. (A) HeLa cells were treated with DMSO or 20 µM Q15 for 24 h. Then, immunofluorescence staining with antibodies against hCAP-G (green) and hCAP-H2 (red) was performed. DNA was stained with DAPI. Bar, 10 µm. (B) At least 100 cells were counted, and the percentages of normal and roundish cells were quantified.

To confirm the interaction between hCAP-G2 and Q15, we performed an *in vitro* binding assay. Whole cell lysates were prepared from SW480 and KMS34 cells and incubated with Q15-immobilized beads for 1 h, followed by immunoblot analyses with specific antibodies (Fig. 4B and C). We found that hCAP-G2 in the cell lysates from both SW480 and KMS34 interacted specifically with Q15-immobilized beads; no interaction with mock beads was detected. Further, we found that SMC2, another subunit of condensin II complex, was also retained specifically on the Q15-immobilized beads. The interaction between hCAP-G2 and Q15 was further investigated by means of a competitive binding assay. Binding of hCAP-G2 to Q15 was inhibited in the presence of 100 µM free Q15, indicating that hCAP-G2 interacts not with the biotin linker, but with Q15 itself (Fig. 4D). We also confirmed that *in vitro* translated-hCAP-G2_262–476_ binds directly to Q15 (data not shown). These results suggest that Q15 binds to the condensin II complex through direct interaction with hCAP-G2 in cell lysates prepared from both SW480 and KMS34.

**Figure 6:**
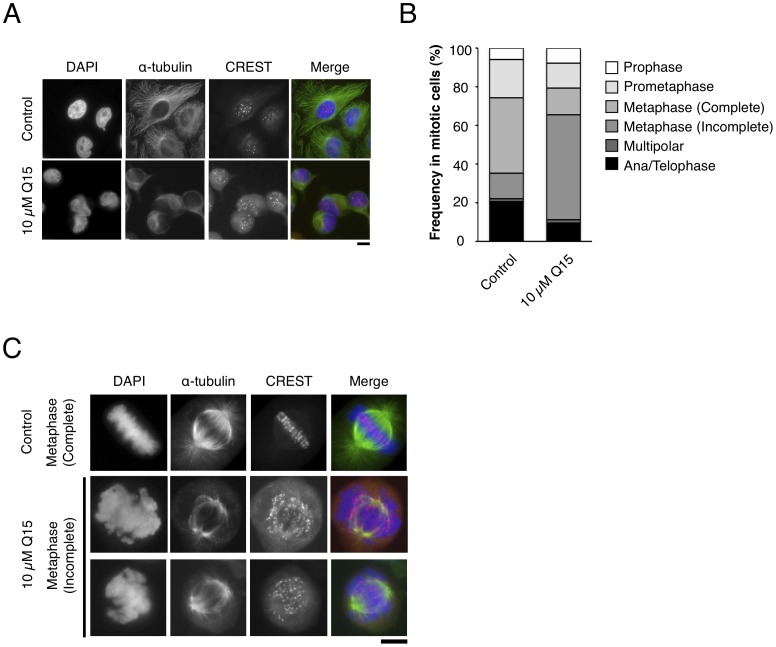
Q15 induces abnormal chromosome segregation. HeLa cells were treated with DMSO (Control) or 10 µM Q15 for 24 h, and immunofluorescence labeling with an antibody against α-tubulin (green) and CREST (red) was performed. DNA was stained with DAPI. (A) Representative images of interphase cells are shown. Bar, 10 µm. (B) Percentages of cells at different stages in mitotic populations are shown. The mitotic stages and alignment defects were judged by DAPI staining. (C) Representative metaphase cells are shown. Q15-treated cells display a dramatically high frequency of metaphase cells with incompletely aligned chromosomes. Bar, 10 µm.

### Q15 Compromises Mitotic Chromosome Segregation and Eventually Induces Apoptosis

Condensins contribute to chromosome assembly and segregation in mitosis [9], [12], [13]. Therefore, we carried out an immunofluorescence analysis to examine the effects of Q15 on the behavior of chromosomes. For this purpose, we selected HeLa cells, since they have a large nucleus and intranuclear structures can be easily observed, whereas KMS34 and SW480 cells are too small for convenient observation of intracellular or intranuclear components. After 24 h treatment with Q15, HeLa cells were labeled with antibodies against hCAP-G and hCAP-H2 to visualize the distribution of condensin I and condensin II, respectively (Fig. 5A). In the Q15-treated cells, we observed about 80% of roundish and swollen chromosomes in which the otherwise distinct localizations of condensin I and condensin II were somewhat obscured (Fig. 5B). The defect in chromosome morphology observed here was reminiscent of, if not identical to, that reported previously in cells depleted of hCAP-G2 [9].

**Figure 7:**
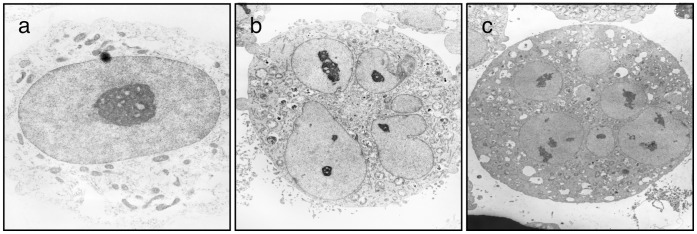
Q15 induces abnormal cell division. KMS34 cells were treated with DMSO (a) or 5 µM Q15 (b and c) for 24 h, then observed with an electron microscope. Micronuclei can be seen in Q15-treated cells.

To examine whether or how Q15 may affect cell cycle progression, we next performed immunofluorescence labeling of cells with an antibody against α-tubulin and CREST, an autoimmune antiserum that recognizes the kinetochore/centromere region. When HeLa cells were treated with Q15 at a final concentration of 10 µM, atrophy of the cytoplasm was observed during interphase (Fig. 6A). Moreover, the frequency of cells with defects in metaphase and anaphase was increased as compared with the control (Fig. 6B). In the metaphase population, more than 50% of mitotic cells showed defects in chromosome alignment (Fig. 6B, metaphase Incomplete). In the ana/telophase population, the frequency of cells with chromosomal bridging and lagging chromosomes among Q15-treated cells was about twice that in untreated cells. As shown in Fig. 6C, the most obvious defect was incompletely aligned chromosomes in metaphase. In these cells, poorly organized chromosomes were scattered and failed to be aligned properly on the metaphase plate. Again, this mitotic phenotype was reminiscent of that previously observed in cells depleted of condensin II [13]. Thus, these results indicate that Q15 compromises proper assembly and segregation of chromosomes, possibly by interfering with the function of condensin II.

We finally examined whether abnormal cell division induced by Q15 affects the nuclear structure of cells. KMS34 cells were treated with 5 µM Q15 for 24 h and observed with an electron microscope (Fig. 7). The Q15-treated cells showed segmented nuclei, while control cells had a single nucleus. These results suggest that interaction of Q15 with hCAP-G2 induces abnormal mitosis, resulting in multinucleated cells.

## Discussion

In this study, we identified a novel anilinoquinazoline derivative Q15 as a potent inhibitor of proliferation of cancer cell lines derived from a variety of tissues. Our results also indicated that Q15 has a more potent antitumor activity than gefitinib, an anilinoquinazoline derivative that has a well-established antitumor effect on recurrent non-small-cell lung tumor [14]. However, unlike gefitnib, Q15 did not inhibit intracellular signaling or the phosphorylation status of ERK1/2, indicating that the mechanism of its antitumor effect is different from that of gefitinib.

We have developed mRNA display using IVV [7], [15], [16] as a simple and totally *in vitro* screening tool for protein-protein [15], protein-peptide [17]–[19], antigen-antibody [20], [21], protein-DNA [22], protein-RNA [23] and protein-drug [24] interactions. In this mRNA display methodology, molecules that interact with target proteins are amplified by RT-PCR, and the amplified sequences are identified by DNA sequencing. Functional domains are easily extracted based on the identified sequences obtained from a randomly primed prey library as a non-biased representation [16], [25]. Bait mRNA templates were prepared using an *in vitro* procedure that makes the previously employed *in vivo* IVV cloning steps unnecessary [7]. Because mRNA display using IVV is an entirely *in vitro* process, both toxic and nontoxic interacting proteins can be characterized. This is a distinct advantage of this method, because toxic proteins are not amenable to characterization by assays that require *in vivo* steps, such as Y2H [26] and TAP-MS [27].

Application of this approach here identified a HEAT-repeat domain of hCAP-G2, which is one of the subunits of condensin II complex, as a Q15 binder that might be related to inhibition of cell proliferation. The condensin II complex is composed of SMC2 and SMC4 ATPases and three auxiliary subunits (hCAP-G2, hCAP-D3 and hCAP-H2) [9], [10], [14], [28]. In binding assay using Q15-immobilized beads, SMC2 was co-precipitated with hCAP-G2, suggesting that Q15 interacts not with monomeric hCAP-G2, but with the holo-complex of condensin II. In condensin I, the HEAT-repeat domains of hCAP-G and hCAP-D2 are suggested to play important roles in their interaction with hCAP-H [29]. Therefore, this might also be the case for the interaction between hCAP-G2 and hCAP-H2. In fact, our data suggested that Q15 binds to HEAT repeats of hCAP-G2, which are thought to facilitate protein-protein interaction [11]. It is therefore possible that Q15 inhibits interactions between hCAP-G2 and hCAP-H2 or between hCAP-G2 and any other chromosomal proteins.

Previous studies showed that siRNA-mediated depletion of hCAP-G2 from HeLa cells results in severe defects in metaphase chromosome morphology [9], chromosome alignment and anaphase chromosome segregation [30]. Other studies also suggested that condensins regulate mitotic chromosome segregation in many different organisms [10], [12], [13]. Here, we have observed distortion of metaphase chromosome morphology, as well as incomplete alignment of metaphase chromosomes, in Q15-treated cells. These features are similar, if not identical, to the phenotypes observed in hCAP-G2-depleted cells, implying that binding of Q15 to hCAP-G2 inhibits condensin II function. Electron microscopic observations further indicated that abnormal cell division induced by Q15 resulted in formation of multinucleated cells.

In conclusion, we have identified a novel anilinoquinazoline derivative Q15 as a growth inhibitor of several intractable cancer cell lines. Using our mRNA display technology, we identified hCAP-G2, a subunit of condensin II complex, which is regarded as a key player in mitotic chromosome condensation, as a Q15 binding partner. We further showed that Q15 induces apoptosis and abnormal chromosome segregation during cell division. Taken together, our results indicate that Q15 may induce mitotic failure in tumor cells by interfering with condensin II function, and this leads to apoptosis. Therefore, inhibition of condensin function could be a novel strategy for the development of antitumor drugs for a range of tumors that are unresponsive to existing drugs.

## Materials and Methods

### Cell Lines

Multiple myeloma cell lines (KMM1, KMS11, KMS26, KMS27, KMS34 and RPMI8226) were generous gifts from Prof. T. Otsuki (Kawasaki Medical College, Kurashiki, Japan) [31] and were maintained in RPMI1640 medium with 10% fetal bovine serum and 1% penicillin/streptomycin. HeLa cells (RIKEN Cell Bank, 2002) and SW480 cells (ATCC, 2005) were maintained in DMEM with 10% fetal bovine serum and 1% penicillin/streptomycin. The sources of other human cancer cell lines are described elsewhere [32]. All cell lines were expanded immediately upon receipt, and multiple vials of low-passage cells were maintained in liquid N_2_. No vial of cells was cultured for more than 3 months. The cells were tested routinely for Mycoplasma and purity. The identification of cell lines was performed based on an STR Multiplex method that uses 9 different loci: D5S818, D13S317, D7S820, D16S539, vWA, TH01, Amelogenin, TPOX and CSF1PO (Powerplex 1.2 system, Promega Corporation) in 2011.

### Compounds

The syntheses of Q15 (6-amino-4(N)-(3-chloro-4-fluorophenyl)-7-[4-[4-phenoxy(phenylcarbamoyl)]piperazin-1-yl]quinazolin-4-amine) and biotinylated Q15 (Q15-Bio) are described in the Methods S1. All other anilinoquinazoline derivatives were synthesized from the corresponding quinazoline derivatives and aniline derivatives. The chemical structures of all synthetic compounds were confirmed by ^1^H-NMR spectroscopy and mass spectrometry.

### Cell Proliferation Assay

The cytotoxicity of each compound was assessed by MTS cell survival assays according to the instructions provided by the manufacturer (Promega, Madison, WI). Briefly, SW480 cells (1×10^4^ cells), KMS11, KMS21, KMS26, KMS28 or KMS34 cells (2.5×10^4^ cells, respectively) were plated in 96-well plates at a density of 20,000 cells per well. Synthetic anilinoquinazoline derivatives were dissolved in DMSO (Sigma) to make 20 mM stock solutions. The stock solutions were then diluted to 0.078–50 µM in medium and distributed in 96-well plates. After 48 or 72 h treatment, 20 µL of the CellTiter 96 AQueous One Solution reagent (Promega) was directly added to each well and the plates were incubated at 37°C for 2 h in 5% CO_2_ in air. The absorbance at 490 nm was read using a Safire™ microplate reader (Tecan, Mannedorf, Switzerland).

### Immunoblot Analysis

Cells were treated with 20 µM Q15 for 48 h followed by lysis in RIPA buffer (50 mM Tris pH 7.6, 150 mM NaCl, 1 mM EDTA, 0.5% sodium deoxycholate, 0.05% SDS, 1% NP-40) containing protease inhibitor cocktail (Nacalai Tesque, Kyoto, Japan). Protein concentrations were determined with a BCA protein assay kit (Thermo Scientific, Waltham, MA). Protein (20 µg) was run on 10 or 15% SDS-PAGE gel and analyzed with antibodies against caspase-9, caspase-3, PARP, p44/42 MAP kinase or phospho-p44/42 MAP kinase (all from Cell Signaling Technology, Beverly, MA). The blots were visualized with ECL chemiluminescence reagents (GE Healthcare, Waukesha, WI).

### *In vivo* Tumor Growth Assay

Animal experiments were approved by the Ethics Committee for Animal Experiments at Keio University (no. 09118-0). *In vivo* tumor inhibition assay was performed as previously described [33] with several modifications. Briefly, 3×10^7^ KMS34 cells were subcutaneously inoculated into 5-week-old male lcr/scid mice (CLEA, Tokyo, Japan). Plasmacytoma developed in 6 to 7 weeks, and when the size of the tumor had reached 50 mm^3^ (day 1), vehicle (saline, 10% DMSO and 1% Tween 80) or 20 mg/kg of Q15 was injected intraperitoneally twice every 3 days. Tumor volumes was calculated according to the following formula: width×length^2^×0.52 [33]. Differences in tumor size on day 14 were evaluated by means of Student’s *t* test. P<0.05 was considered to indicate statistical significance.

### Histopathologic Examination

Histopathologic analysis was performed as previously described [33] with several modifications. When the size of subcutaneous tumors reached 50 mm^3^, vehicle or 20 mg/kg Q15 was injected intraperitoneally twice every 3 days. After 14 days, the mice were killed and the tumors were isolated. Tumor samples were fixed with 10% formalin and embedded in paraffin. Sections were stained with hematoxylin and eosin.

### mRNA Display Selection

Total RNA from SW480 cells was extracted with an RNeasy mini kit (Qiagen, Valencia, CA) and purified with a mTRAP mRNA isolation kit (Active Motif, Carlsbad, CA). A cDNA library was prepared as previously described [15]. The resulting cDNA library derived from KMS34 cells was transcribed using a RiboMAX large-scale RNA production system-SP6 (Promega) in a total volume of 20 µL containing 80 mM HEPES-KOH, pH 7.5, 2 mM spermidine, 40 mM DTT, 32 mM MgCl_2_, 5 mM each of ATP, CTP, and UTP, 1 mM GTP, 5 mM m^7^G(5′)ppp(5′)G RNA capping analog (Invitrogen, Carlsbad, CA), 1 pmol of KMS34 cDNA library and SP6 RNA polymerase. The resulting RNA was purified with an RNeasy mini kit and ligated with a PEG-puromycin spacer [p(dCp)_2_-T(fluorescent)p-PEGp-(dCp)_2_-puromycin] [15] using T4 RNA ligase (Takara) for 15 h at 15°C in a total volume of 50 µL containing 50 mM Tris-HCl, pH 7.5, 10 mM MgCl_2_, 12 mM DTT, 1.4 mM ATP, 5% DMSO, 0.002% BSA, 40 U of RNase inhibitor (Toyobo, Osaka, Japan), 0.2 mM PEG-puromycin spacer, 0.6 mM PEG2000 (Nacalai Tesque), 50 pmol of RNA, and 200 U of T4 RNA ligase. The resulting RNA-PEG-puromycin library was purified with the RNeasy mini kit. *In vitro* translation was performed in a total volume of 250 µL containing 25 pmol of the RNA-PEG-puromycin library, 50 µL of wheat germ cell-free extract (Zoegene, Kanagawa, Japan), 100 µg of creatine kinase, and 50 µL of a translation buffer (Zoegene) for 1 h at 26°C. The reaction mixture was added to Q15-Bio immobilized on Magnotex SA beads (Takara, Otsu, Japan) pre-equilibrated with IPP150 (10 mM Tris-HCl pH 8.0, 150 mM NaCl and 0.1% NP-40), and mixed on a rotator for 1 h at 4°C. The beads were washed five times with IPP150 and used as the template for RT-PCR with a OneStep RT-PCR kit (Qiagen) using primers (5′- GGAAGATCTATTTAGGTGACACTATAGAACACAACAACAACAAACAACAACAAAAT-G-3′) and (5′-TTTTTTCTTGTCGTCATCGTCCTTGTAGTC-3′). The RT-PCR product was used for the next round of selection as described above. After five rounds of selection, the RT-PCR product was cloned using a PCR cloning kit (Qiagen) and sequenced with an ABI PRISM 3100 Genetic Analyzer (Applied Biosystems, Foster City, CA).

### *In vitro* Binding Assay

SW480 or KMS34 cells were lysed with lysis buffer (50 mM Tris-HCl pH 7.5, 150 mM NaCl, 1% NP-40 and 0.1% DOC) containing protease inhibitor cocktail (Nacali tesque). The whole cell lysates were added to Q15-immobilized beads and mixed on a rotator for 1 h at 4°C in the absence or presence of 100 µM free Q15. The beads were washed five times with lysis buffer, followed by vortexing to elute the bound molecules. The resulting eluate was loaded on a 10% SDS-PAGE gel, and analyzed by immunoblot analysis with antibodies against LUZP5 (Bethyl Laboratories, Montgomery, TX) and SMC2 (Bethyl Laboratories).

### Immunofluorescence Assay

HeLa cells on coverslips were treated with 5 µM Q15 for 24 h. Colcemid was then added at a final concentration of 0.02 µg/mL, and incubation was continued for another 3 h. Mitotic cells were collected by tapping the coverslips, followed by centrifugation at 1,000*g* for 5 min (Thermo/Shandon, Cyospin 4). The collected cells were fixed with 2% PFA in PBS at room temperature for 15 min, and treated with 0.5% Triton X-100 in PBS at room temperature for 5 min. The cells on coverslips were stained with an antibody against α-tubulin (Sigma, T9026/DM1A) and CREST [34], followed by Alexa488-labelled goat anti-mouse IgG and Alexa594-labelled goat anti-human IgG (Invitrogen, Carlsbad, CA). The mitotic spread cells were stained with antibodies against hCAP-G conjugated with biotin [30], [35] and hCAP-H2 [9], followed by Alexa488-labelled streptavidin and Alexa594-labelled donkey anti-rabbit IgG (Invitrogen).

### Electron Microscopic Observation

KMS34 cells were treated with 5 µM Q15 for 24 h. The cells were fixed in 2.5% glutaraldehyde in 100 mM sodium cacodylate (pH 7.2) for 1 h, washed, postfixed in 1% osmium tetroxide for 2 h, stained en bloc with uranyl acetate, dehydrated in a series of graded ethanol solutions, and embedded in an Epon/Araldite mixture. Ultrathin sections were stained with lead citrate and examined under an electron microscope (Nippon Denshi EX-200, Tokyo, Japan).

## Supporting Information

Methods S1 **Synthesis of Q15 and biotinylated Q15.**
(PDF)
