# Supplementary material for: An Anilinoquinazoline Derivative Inhibits Tumor Growth through Interaction with hCAP-G2, a Subunit of Condensin II
Source: PLoS One. 2012 Sep 13;7(9):e44889. doi: 10.1371/journal.pone.0044889 (PMC3441599; doi:10.1371/journal.pone.0044889)
Supplement: Methods S1 — Synthesis of Q15 and biotinylated Q15. (PDF) [file pone.0044889.s001.pdf]

Methods S1

Q15 (4) was synthesized from (7-chloro-6-nitro-4-quinazolinyl)-(3-chloro-4- fluorophenyl) amine (1) in 3 steps as follows.

Synthesis of [6-Nitro-7-(1-piperazino)-4-quinazolinyl]-(3-chloro-4-fluorophenyl)amine (2)

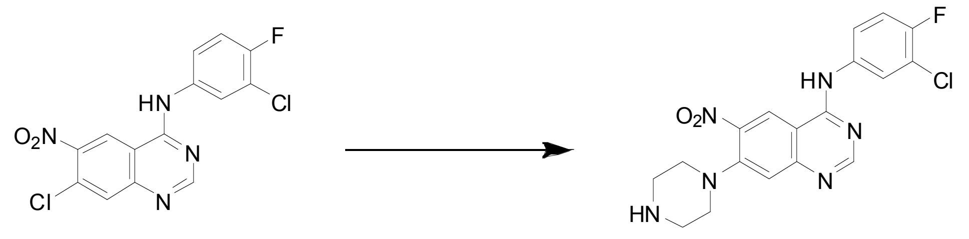

1 **2**

(7-chloro-6-nitro-4-quinazolinyl)-(3-chloro-4-fluorophenyl)amine (1) was synthesized according published procedure[1]. Piperazine (103.9 mg, 1.21 mmol) in n-butanol (6 ml) was added to 1 (212.9 mg, 0.603 mmol) and the mixture was refluxed for 12h. After evaporation of the solvent, the residue was treated with ethyl acetate (10 ml) and the resulting precipitate was collected on a filter as 2 (142 mg, 58%).
1H NMR (DMSO-d6, 400MHz) δ ppm: 9.24 (s, 1H), 8.65 (s, 1H), 8.19 (m, 1H), 7.81 (m, 1H), 7.47 (t, 1H), 7.38 (s, 1H), 3.34 (br s, 1H), 3.24 (m, 4H), 3.08 (m, 4H).

[1]Kitano Y, Kawahara E, Suzuki T, Abe D, Nakajou M, Ueda N. Quinazoline derivatives. United States Patent Application 20070265260, November 15, 2007.

Synthesis of [6-Nitro-7-[4-(p-phenoxyphenyl)carbamoyl]-1-piperazino]-4-quinazolinyl]-(3-chloro-4-fluorophenyl)amine (3)

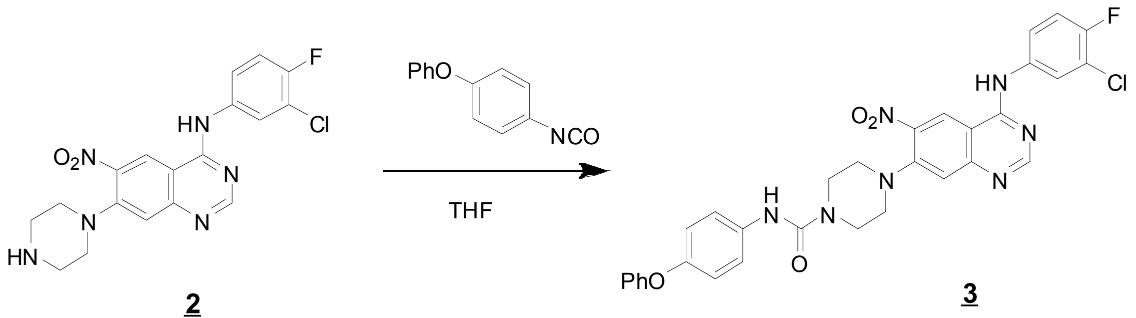

2 **3**

To a solution of 3 (199mg, 0.32 mmol) in glacial acetic acid (4 ml), was added reduced iron powder (1 g, 17.9 mmol) and the suspension was stirred for 12 h at room temperature. The reaction mixture was filtered to remove the iron residue which was washed with ethyl acetate (30 ml). The filtrate was concentrated under reduced pressure. To the residue was added ethyl acetate (30 ml) and 2M KOH (20 ml) and the organic layer was partitioned. The water layer was further extracted with ethyl acetate (3 x 25 ml). The combined organic layers were washed with brine (2 x 25 ml) and water (3 x 25 ml), dried (Na_2SO_4) and concentrated under reduced pressure to obtain 4 (Q15, 179 mg. 95%).
1H NMR (DMSO-d6, 400 MHz) δ ppm: 9.44 (s, 1H), 8.65 (s, 1H), 8.37 (s, 1H), 8.18 (dd,1H), 7.80 (m, 1H), 7.33-7.55 (m, 7H), 7.23 (s, 1H), 6.96 (m, 2H), 5.31 (s, 2H), 3.46 (s, 8H).

Synthesis of [6-Amino-7-[4-(p-phenoxyphenyl)carbamoyl]-1-piperazino]-4-quinazolinyl]-(3-chloro-4-fluorophenyl)amine (4)

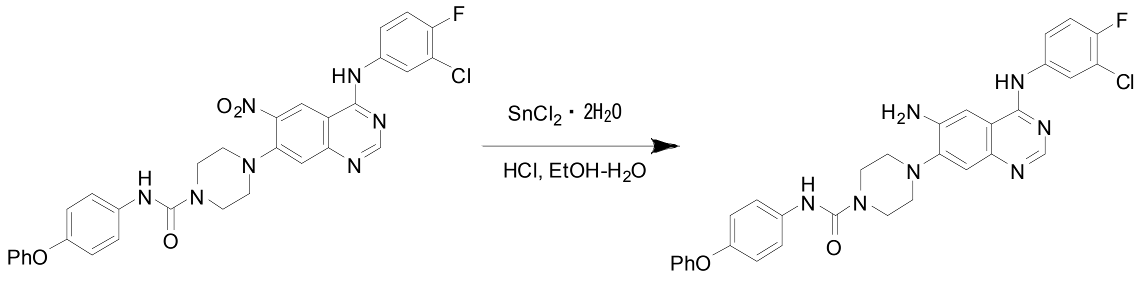

3 **4**

To a solution of 3 (199mg, 0.32 mmol) in glacial acetic acid (4 ml), was added reduced iron powder (1 g, 17.9 mmol) and the suspension was stirred for 12 h at room temperature. The reaction mixture was filtered to remove the iron residue which was washed with ethyl acetate (30 ml). The filtrate was concentrated under reduced pressure. To the residue was added ethyl acetate (30 ml) and 2M KOH (20 ml) and the organic layer was partitioned. The water layer was further extracted with ethyl acetate (3 x 25 ml). The combined organic layers were washed with brine (2 x 25 ml) and water (3 x 25 ml), dried (Na_2SO_4) and concentrated under reduced pressure to obtain 4 (Q15, 179 mg. 95%).
1H NMR(DMSO-d6, 400 MHz)δ ppm: 9.44 (s, 1H), 8.65 (s, 1H), 8.37 (s, 1H), 8.18 (dd,1H), 7.80 (m, 1H), 7.33-7.55 (m, 7H), 7.23 (s, 1H), 6.96 (m, 2H), 5.31 (s, 2H), 3.46 (s, 8H).

Synthesis of biotinylated Q15 (Q15-Bio)

Biotinylaed Q15 (Q15-Bio) and activated biotin in 2 steps was synthesized from Q15 as follows.

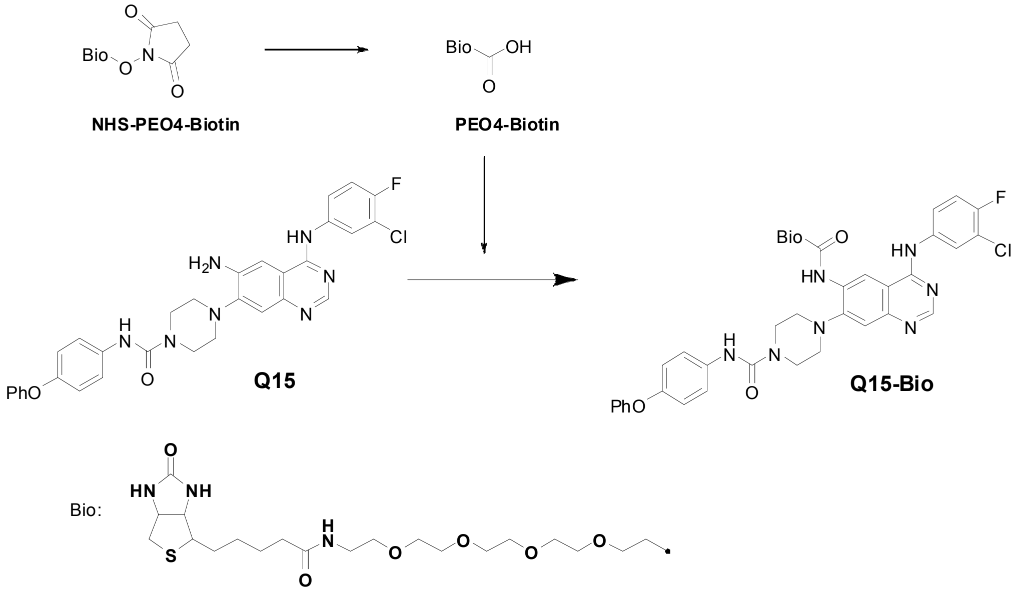

To a solution of NHS-PEO_4-Biotin(10 mg, 17 μmol, Thermo Scientific) in water (0.1 ml), 1M NaOH (19 ml) was added. The mixture was stirred for 2 h at room temperature and then 1M HCl (22 ml) was added to the above mixture. The reaction mixture was concentrated under reduced pressure and the residue was co-evaporate with anhydrous pyridine (5 ml) twice to obtain HOOC-PEO4-Biotin. To a solution of 4 (Q15, 9.9mg, 17 μmol) in pyridine (0.5 ml), PCl3 (2.3 mg, 17 μmol) was added. The mixture was heated for 1 h at 80 °C, and then the above HOOC-PEO4-Biotin in pyridine (0.1 ml) was added to the mixture. The reaction mixture was heated for 12 h at 80 °C and then water (1 ml) was added to the reaction mixture. The mixture was concentrated to dryness. The residue was resolved water (0.1 ml) and purified by C-18 reverse-phase HPLC to obtain Q15-Bio (2 mg, 11%). Mass (ESI): m/z = 1057 (M+1)

HPLC conditions

Colunm: CombiPrep Pro C18 S-5 mm, 20 mm diameter × 50 mm length (YMC, Kyoto, Japan)
Solvent A: Water (0.05 % TFA)
Solvent B: MeCN (0.05 % TFA)
Flow rate: 25.0 ml/min
Linear Gradient:

Time (min)	A (%)	B (%)
0.0	99	1
1.0	99	1
4.5	60	40
7.6	10	90
9.2	10	90
10.0	99	1
11.0	99	1
